# Self-Powered Electrospun Composite Nanofiber Membrane for Highly Efficient Air Filtration

**DOI:** 10.3390/nano10091706

**Published:** 2020-08-29

**Authors:** Zungui Shao, Jiaxin Jiang, Xiang Wang, Wenwang Li, Liang Fang, Gaofeng Zheng

**Affiliations:** 1 Department of Instrumental and Electrical Engineering, Xiamen University, Xiamen 361102, China; 35120191151216@stu.xmu.edu.cn (Z.S.); jiangjx@xmu.edu.cn (J.J.); 2 Shenzhen Research Institute of Xiamen University, Shenzhen 518000, China; 3 School of Mechanical and Electrical Engineering, Tan Kah Kee College of Xiamen University, Zhangzhou 363105, China; 4 School of Mechanical and Automotive Engineering, Xiamen University of Technology, Xiamen 361024, China; wx@xmut.edu.cn (X.W.); xmlww@xmut.edu.cn (W.L.)

**Keywords:** self-powered membrane, electrospun nanofiber, air filtration, electrostatic adsorption, triboelectric effect

## Abstract

Highly efficient air filtration with low pressure drop is the key to air purification. In this work, a self-powered electrospun nanofiber membrane with an electrostatic adsorption effect was prepared to improve the filtration efficiency of micro/nano particles. The composite membrane was comprised of polyvinyl chloride (PVC) nanofibers and polyamide-6 (PA6) nanofibers. The triboelectric effect between the two adjacent nanofiber membranes generated electrostatic charges under the action of air vibration, by which the electrostatic adsorption with the same pressure drop was enhanced. The electrostatic voltage on the self-powered nanofiber membrane was 257.1 mV when the flow velocity was 0.1 m/s. For sodium chloride (NaCl) aerosol particles with a diameter of 0.3 μm, the removal efficiency of the self-powered composite nanofiber membrane was 98.75% and the pressure drop was 67.5 Pa, which showed a higher quality factor than the membrane without electrostatic charges. This work provides an effective way to improve the filtration performance of air filter membranes.

## 1. Introduction

Air filtration has become the focus of global environmental protection [1,2,3,4]. The existence of fine particulate matter (PM) has posed a great threat to public health [5,6]. It is required urgently to realize the highly efficient filtration of ultrafine particles. The novel fabrication technology of filtration membranes has, thus, become a research hotspot in the field of environmental pollution control [7,8]. 

Electrospinning is a versatile method used to prepare nanofibers [9,10,11,12]. Due to the advantages of their ultrafine diameter, small characteristic size, large specific surface area and high porosity, electrospun nanofiber membranes are promising candidate for particulate matter removal [13,14,15,16]. On the other hand, electrospun nanofibers can store charges to increase the electrostatic adsorption capacity of particles with diameters less than or equal to 0.3 μm [17,18,19,20], which also enhance the purification effect for bacteria and viruses. A high-performance nanofiber membrane with high filtration efficiency and low pressure drop is desired for industrial applications [21,22].

At present, electrified filtration membranes have exhibited an excellent potential to increase their electrostatic charges so as to improve filtration performance [23]. Adding an electret is a common way to improve the electrification effect to enhance electrostatic adsorption capacity [24]. Li et al. [25] added hydrophobic silica (SiO_2_), boehmite, barium titanite (BaTiO_3_) and silicon nitride (Si_3_N_4_) nanoparticles into a polyetherimide (PEI) solution to prepare electrospun nanofibers, which performed a stable surface potential. The filtration efficiency was over 99.99% for 0.3 μm NaCl aerosol particles, and the pressure drop was just 61 Pa. Wang et al. [26] used polytetrafluoroethylene (PTFE) nanoparticles as an electret to maintain the charges on the surface of polyvinylidene fluoride (PVDF) nanofibers to achieve a filtration efficiency of 99.972% and a pressure drop of 57 Pa for 0.3–0.5 μm aerosol particles. As the charges on the surface of the nanofibers will disappear sometime after the electret treatment and decrease the filtration efficiency, the electrified-charge stability of nanofiber membranes remains a challenge for highly efficient filtration.

Self-powering is a new method to promote the durability of charged filtration nanofiber membranes [27] which can generate charges to capture particulate matter during the filtration process [28]. According to the generation principles of electrical charges, these methods can be divided into various catalogues including piezoelectric [29], electromagnetic [30] and triboelectric [31]. The triboelectric nanofiber membrane is based on the principle of friction electricity generation. Gu et al. [32] proposed an external triboelectric nanogenerator to charge the nanofiber membrane, by which the average filtration efficiency of particles with a diameter of 53.3 nm reached 89.9%. Liu et al. [21] developed a novel self-powered electrostatic adsorption face mask based on a PVDF electrospun nanofiber membrane, which was driven by respiration. The filtration efficiency remained up to 86.9% after being continually worn for 240 min and over a 30-day period. However, due to its complex structure, it is difficult to expand the application fields of the triboelectric filtration mask. 

In this paper, a novel method was developed to fabricate a self-powered electrospun composite nanofiber membrane. Different electronegative materials were utilized to prepare the nanofibers, on which charges can be generated on the membrane surface based on the triboelectric effect. The charge generation capacity of the composited nanofiber membrane and the effects on the filtration performance were discussed.

## 2. Experimental Details 

### 2.1. Preparation of Self-Powered Nanofiber Membrane

Polyvinyl chloride (PVC) and polyamide-6 (PA6) of different electro polarities [33] were used to prepare the composite nanofiber membrane. PA6 powder (*M*_w_ = 100,000 g/mol) was dissolved in 88% formic acid with a mass concentration of 15 wt%. PVC powder (*M*_w_ = 80,000 g/mol) was dissolved in a mixture solvent of *N,N*-dimethylformamide (DMF) and tetrahydrofuran (THF) (*v*:*v* = 7:3) with a mass concentration of 10 wt%. The PA6 powder and the PVC powder were purchased from Guangdong TPL Chemical Co. Ltd, Guangdong, China. The formic acid (88%), DMF and THF were purchased from Sinopharm Chemical Reagent Co. Ltd, Shanghai, China.

The PA6 nanofiber membrane was prepared at the distance between the spinneret (Wenzhou Hongxing Medical Equipment Plant, Wenzhou, China) and the collector (Xiamen Nalai Technology Co., Ltd, Xiamen, China), and the applied voltage and supply rate of the solution of 15 cm were 20 kV and 0.1 mL/h, respectively. The PVC nanofiber membrane was prepared at the distance between the spinneret and the collector, and the applied voltage and supply rate of the solution of 13 cm were 15 kV and 0.5 mL/h, respectively. The inner diameter of the spinneret was 0.26 mm. The temperature and relative humidity during the electrospinning process were controlled at 25 °C and 50%, respectively. A grounded electrode covered with gauze was used as the collector for the PA6 and PVC electrospun nanofibers. Both of the nanofiber membranes were hot-pressed at a temperature of 50 °C to evaporate the excess solvent and to gain solid nanofibers. 

Then, the self-powered membrane was prepared by directly combining the PA6 nanofiber membrane and the PVC nanofiber membrane, while the non-charged membrane was fabricated by combining the PA6 nanofiber membrane and the PVC nanofiber membrane with gauze between them.

In the experiments, the electrospinning time was set to 15 min, 30 min, 45 min, 60 min and 75 min. The corresponding average thickness of the PA6 membrane was 2.3 μm, 4.5 μm, 6.8 μm, 9.0 μm and 11.3 μm, respectively, and that of the PVC membrane was 1.5 μm, 3.1 μm, 4.5 μm, 5.9 μm and 7.5 μm, respectively. The basis weights of the prepared composite membranes were 0.25 g/m^2^, 0.49 g/m^2^, 0.74 g/m^2^, 0.99 g/m^2^ and 1.2 g/m^2^, respectively.

### 2.2. Filtration Theory of Self-Powered Nanofiber Membrane

**Figure 1:**
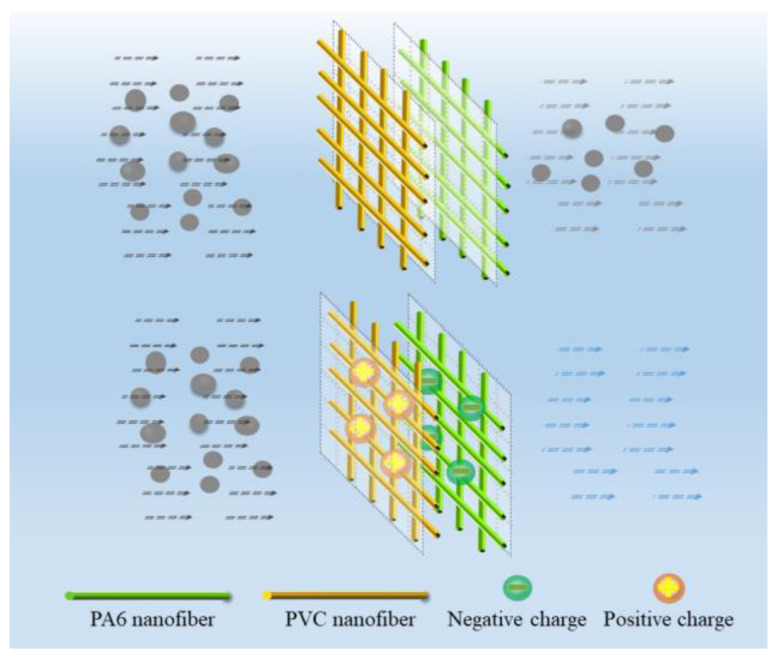
Schematic diagram for the air filtration process of the self-powered composite membrane.

The schematic diagram of the self-powered nanofiber membrane for particle filtration is illustrated in Figure 1. The PA6 nanofiber membrane and the PVC nanofiber membrane were combined directly to generate charges through surface friction between the two nanofiber membranes. Attributed to the vibration of the air flow, an equal amount of heterogeneous charges was generated on the contact surfaces, by which the PVC membrane surface was negatively charged and the PA6 membrane was positively charged. In this way, the charged nanofiber membrane could display a better electrostatic adsorption performance for particle filtration than that without surface contact. 

### 2.3. Testing of Self-Powered Nanofiber Membrane

**Figure 2:**
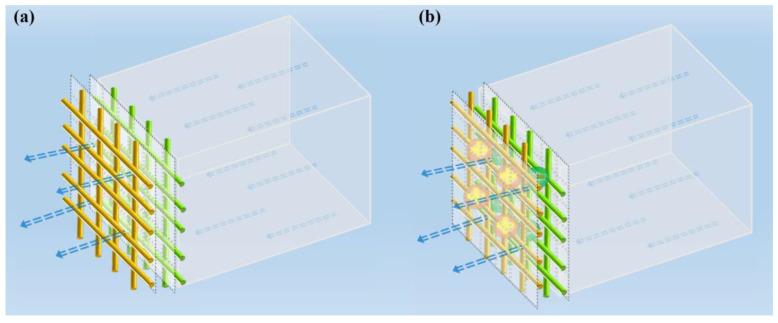
Test schematic diagram of the composite nanofiber membranes: (**a**) non-charged membrane and (**b**) self-powered membrane.

The test schematic diagram of the self-powered nanofiber membrane is shown in Figure 2. The sodium chloride (NaCl) aerosol particles with a diameter of 0.3 μm were generated with an aerosol particle generator and passed through each membrane at a constant airflow velocity of 0.1 m/s and 0.15 m/s. The effective area of the nanofiber membrane was 10 cm^2^. The charges at the interface between the PVC and PA6 membranes were measured with a hand-held electrostatic meter (FMX-003, SIMCO-ION, Kobe, Japan). Then, two copper electrodes were attached to the non-contact sides of the two different membranes to measure their respective voltages. The voltages were measured with an oscilloscope (ZDS1104, Keysight Technology (China) Co., Ltd, Beijing, China).

The number of particles at the inlet and outlet was counted by two laser photometers. The pressure drop (Δp) was measured by two laser photometers and a pressure transmitter. The filtration efficiency (FE) was calculated by Equation (1) and the quality factor (QF) was used to evaluate the filtration performance of the membrane, which can be obtained by Equation (2).

(1) $$FE(\%)=1-\frac{N_{o}}{N_{i}}$$

(2) $$QF\left(Pa^{-1}\right)=-\frac{\ln\left(1-FE\right)}{\Delta p}$$

where, the number of particles at the inlet and the outlet was *N*_i_ and *N*_o_, respectively. 

## 3. Results and Discussion

### 3.1. Electrospun Nanofibers

**Figure 3:**
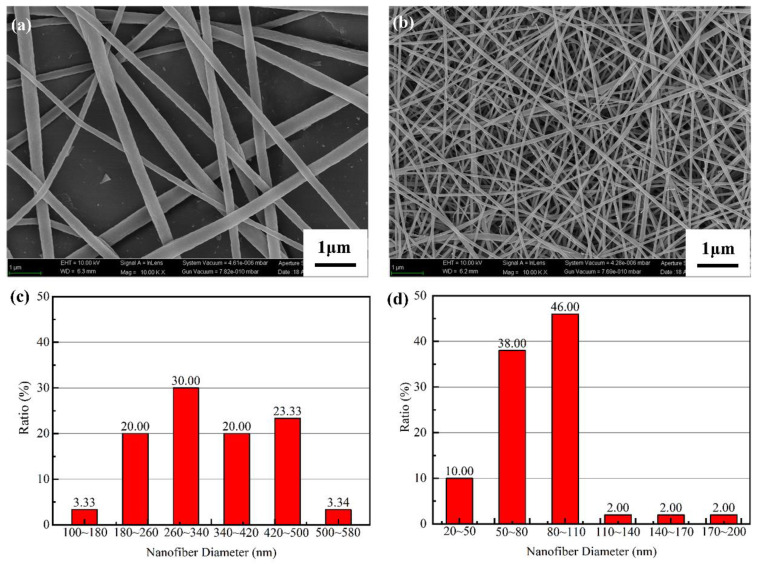
Nanofiber membranes. (**a**) scanning electron microscope (SEM) images of polyamide-6 (PA6) nanofibers; (**b**) SEM images of polyvinyl chloride (PVC) nanofibers, where magnification was 10,000×; (**c**) diameter distribution of the PA6 nanofibers; (**d**) diameter distribution of the PVC nanofibers.

The scanning electron microscope (SEM) (SUPRA 55 SAPPHIRE, Carl Zeiss AG, Jena, Germany) images of the electrospun PA6 nanofibers and PVC nanofibers are shown in Figure 3. The PA6 nanofibers (Figure 3a) and PVC nanofibers (Figure 3b) have a smooth surface, uniform diameter, and no droplets, which enables full friction between them and enables a better mechanical filtration ability. The diameter distribution of the nanofibers is shown in Figure 3c,d, where the average diameter of the PA6 nanofibers was 350 nm, and that of the PVC nanofibers was 100 nm.

### 3.2. Measurement of Electrical Charge

**Figure 4:**
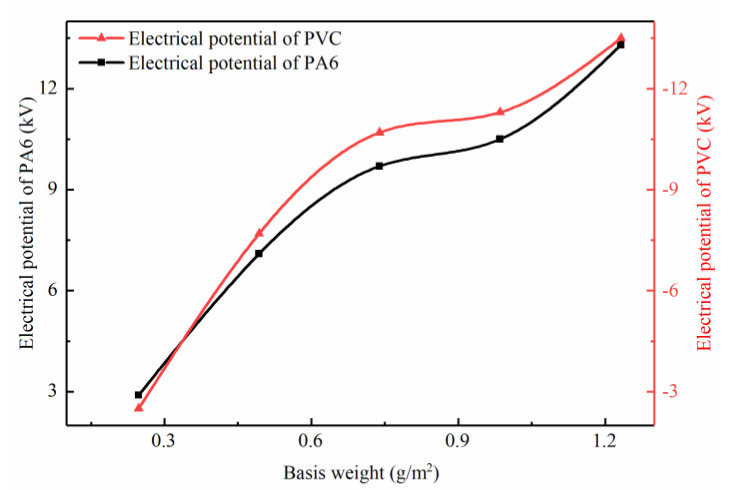
The electrical potential of the nanofiber membrane under an airflow velocity of 0.15 m/s.

The PVC nanofiber had a strong electronegativity, while the PA6 nanofiber had a weak electronegativity. During the filtration process, the vibration can stimulate the triboelectric effect on the interface of different nanofiber membranes to generate charges. The measurement result of the electrical charges on the interface of the PA6 nanofiber membrane and the PVC nanofiber membrane is shown in Figure 4. After ventilation for 3 min under an airflow velocity of 0.15 m/s, both of the charges on the surface of the PA6 and PVC nanofiber membrane raised with the increase in the basis weight. The surface of the PA6 nanofiber membrane was positively charged, while the surface of the PVC nanofiber membrane was negatively charged, which was consistent with the electronegativity. The maximum surface potential could reach about ±12 kV. In addition, the surface charge of the PA6 nanofiber membrane was basically the same as that of the PVC nanofiber membrane, which conformed to the charge transfer principle of triboelectrification.

### 3.3. Measurement of Self-Powered Voltage

**Figure 5:**
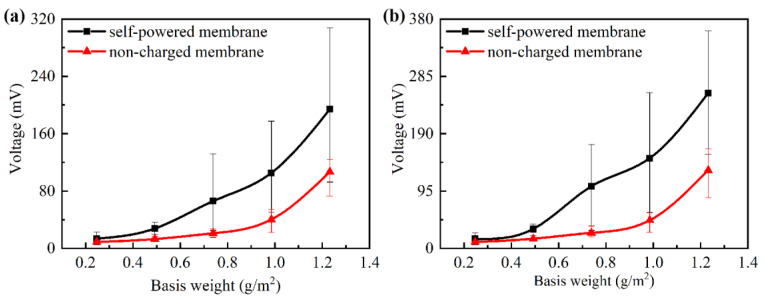
The measured voltage on the two sides of the composite membranes of different combinations under different airflow velocities: (**a**) 0.1 m/s and (**b**) 0.15 m/s.

During the filtration process, the PVC and PA6 nanofiber membranes continuously combined and separated when the airflow passed through the composite membrane, and a dynamic voltage was generated by the friction of the air on the nanofibers and the triboelectric effect between the PVC and PA6 nanofiber membranes. The voltages on the two sides of the composite membrane of different combinations under an airflow velocity of 0.1 m/s and 0.15 m/s are shown in Figure 5. With the increase in the basis weight of the composite membrane, the friction effect was enhanced, so the self-powered voltage was increased. Compared with the non-charged membrane, the self-powered membrane had a larger voltage value, which indicated that the friction between the PVC and PA6 nanofiber membranes had a considerable power generation effect. In addition, with the increase in the airflow velocity, the vibration of the composite membrane was enhanced, and the separation distance between the PA6 and PVC nanofiber membranes increased. According to the working principle of friction nanogenerator [34], the increase in separation distance could improve the voltage between the PA6 and PVC nanofiber membranes.

### 3.4. Filtration Performance

**Figure 6:**
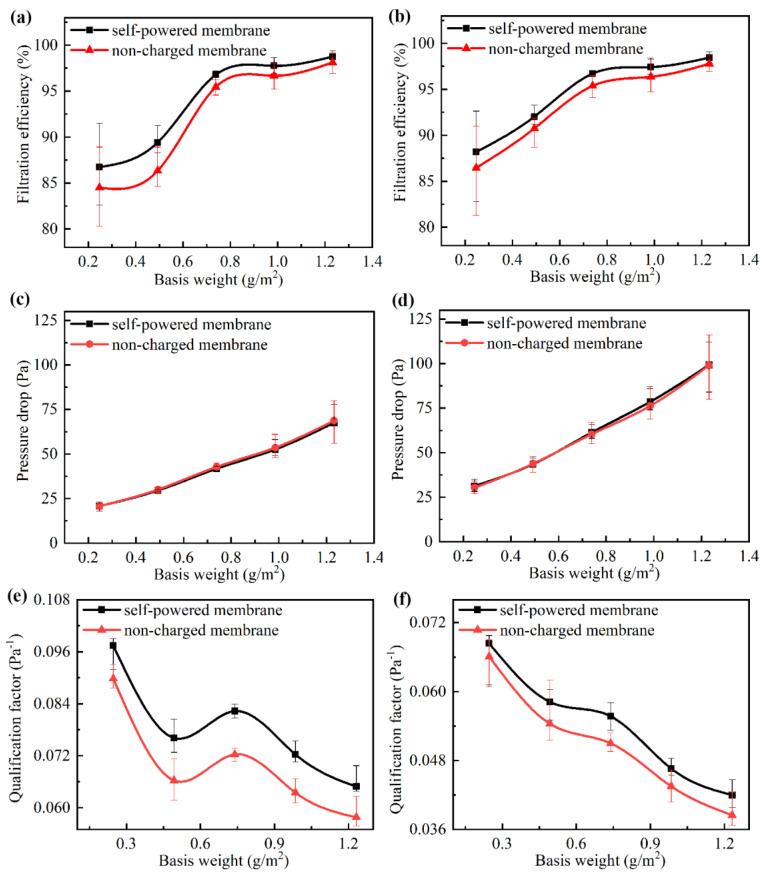
The filtration efficiency (*FE*) of the composite membranes under different airflow velocities: (**a**) 0.1 m/s and (**b**) 0.15 m/s. The pressure drop in the composite membranes under different airflow velocities: (**c**) 0.1 m/s and (**d**) 0.15 m/s. The quality factor (*QF*) of the composite membranes under different airflow velocities: (**e**) 0.1 m/s and (**f**) 0.15 m/s.

The results of testing the filtration efficiency and pressure drop under an airflow velocity of 0.1 m/s and 0.15 m/s are shown in Figure 6. With the increase in the basis weight, the mechanical *FE* of both of the composite membranes increased. The self-powered membrane showed a higher *FE* due to its considerable triboelectricity, which increased its particle-capturing ability without increasing the pressure drop. Due to the low mechanical filtration efficiency of the two composite membranes with a low basis weight, the triboelectric effect on the improvement of filtration efficiency was more obvious. The *FE* of the self-powered membrane was 2.2% higher than that of the non-charged membrane under the condition of a low basis weight. Finally, it can be seen that the average filtration efficiency of the self-powered membrane was 1.5% higher than that of the non-charged membrane; the *FE* of the self-powered membrane with the basis weight of 1.2 g/m^2^ was 98.75% and the pressure drop was only 67.5 Pa.

The results of the *QF* under an airflow velocity of 0.1 m/s and 0.15 m/s are shown in Figure 6e,f. With its higher filtration efficiency and guaranteed pressure drop, the self-powered membrane exhibited a better comprehensive filtration capacity. With the increase in the basis weight, the mechanical filtration efficiency and the pressure drop of the two groups of composite membranes both increased. However, as the improvement in *FE* was insufficient to make up the increase in pressure drop, the *QF* decreased. When the basis weight of the composite membrane was less than 0.74 g/m^2^, the low density and porosity of the nanofiber membrane made the filtration efficiency lower. When the basis weight continued to increase, the porosity and density of the composite membrane also increased, and so the filtration efficiency increased significantly, while the pressure drop increased linearly, leading to a certain incline of *QF* at 0.74 g/m^2^. 

In the case of the same airflow velocity and basis weight, the *QF* of the self-powered membrane was higher than that of the non-charged membrane, which shows that the self-powered composite membrane can effectively balance the *FE* and the pressure drop, so as to improve the filtration performance of the composite membrane. The highest *QF* of the self-powered membrane was 0.0974 Pa^-1^, which was 8.46% higher than that of the non-charged membrane, which was only 0.0898 Pa^-1^.

## 4. Conclusions

Herein, PA6 and PVC nanofiber membranes were prepared by electrospinning and bonded to a self-powered air filtration composite membrane. Under the action of airflow, the PA6 and PVC nanofiber membranes with opposite electronegativity generated electricity by friction so as to electrostatically adsorb the ultrafine particles in the air. Through the self-powered effect of the composite membrane, the filtration efficiency was improved while the pressure drop was kept at a constant level. Furthermore, the PA6 and PVC nanofiber membranes had a certain mechanical filtration ability. Under the double effects of mechanical filtration and electrostatic adsorption, the air filtration performance was further improved. With the self-powered composite nanofiber membrane, the *FE* could reach up to 98.75% and the highest *QF* was 0.0974 Pa^-1^. In summary, this work provides a way to balance the filtration efficiency and pressure drop of air filtration based on the principle of self-powered. 

## References

[1] Sun Y., Zhuang G., Wang Y., Han L., Guo J., Dan M., Zhang W., Wang Z., Hao Z. (2004). The air-borne particulate pollution in beijing—concentration, composition, distribution and sources. Atmos. Environ..

[2] Li K., Lin B. (2015). Impacts of urbanization and industrialization on energy consumption/co2 emissions: Does the level of development matter?. Renew. Sust. Energ. Rev..

[3] Luo W., Phan H.V., Xie M., Hai F.I., Price W.E., Elimelech M., Nghiem L.D. (2017). Osmotic versus conventional membrane bioreactors integrated with reverse osmosis for water reuse: Biological stability, membrane fouling, and contaminant removal. Water Res..

[4] Canalli Bortolassi A.C., Guerra V.G., Aguiar M.L., Soussan L., Cornu D., Miele P., Bechelany M. (2019). Composites based on nanoparticle and pan electrospun nanofiber membranes for air filtration and bacterial removal. Nanomaterials.

[5] Metia S., Ha Q.P., Duc H.N., Scorgie Y. (2020). Urban air pollution estimation using unscented kalman filtered inverse modeling with scaled monitoring data. Sustain. Cities Soc..

[6] Wang X., Xiang H., Song C., Zhu D., Sui J., Liu Q., Long Y. (2020). Highly efficient transparent air filter prepared by collecting-electrode-free bipolar electrospinning apparatus. J. Hazard. Mater..

[7] Wang Z., Zhang Y., Ma X.Y.D., Ang J., Zeng Z., Ng B.F., Wan M.P., Wong S.-C., Lu X. (2020). Polymer/mof-derived multilayer fibrous membranes for moisture-wicking and efficient capturing both fine and ultrafine airborne particles. Sep. Purif. Technol..

[8] Nanaki E.A., Koroneos C.J., Roset J., Susca T., Christensen T.H., De Gregorio Hurtado S., Rybka A., Kopitovic J., Heidrich O., López-Jiménez P.A. (2017). Environmental assessment of 9 european public bus transportation systems. Sustain. Cities Soc..

[9] Li W., Zheng G., Wang X., Zhang Y., Li L., Wang L., Wang H., Sun D. (2011). Directly electrospun ultrafine nanofibres with cu grid spinneret. J. Phys. D-Appl. Phys..

[10] Xue J., Wu T., Dai Y., Xia Y. (2019). Electrospinning and electrospun nanofibers: Methods, materials, and applications. Chem. Rev..

[11] Zheng G., Jiang J., Wang X., Li W., Liu J., Fu G., Lin L. (2020). Nanofiber membranes by multi-jet electrospinning arranged as arc-array with sheath gas for electrodialysis applications. Mater. Des..

[12] Zheng G., Jiang J., Wang X., Li W., Zhong W., Guo S. (2018). Self-cleaning threaded rod spinneret for high-efficiency needleless electrospinning. Applied Physics A.

[13] Zhang S., Liu H., Tang N., Zhou S., Yu J., Ding B. (2020). Spider-web-inspired pm0.3 filters based on self-sustained electrostatic nanostructured networks. Adv. Mater..

[14] Kadam V., Kyratzis I.L., Truong Y.B., Schutz J., Wang L., Padhye R. (2019). Electrospun bilayer nanomembrane with hierarchical placement of bead-on-string and fibers for low resistance respiratory air filtration. Sep. Purif. Technol..

[15] Sundarrajan S., Tan K.L., Lim S.H., Ramakrishna S. (2014). Electrospun nanofibers for air filtration applications. Procedia Eng..

[16] Balamurugan R., Sundarrajan S., Ramakrishna S. (2011). Recent trends in nanofibrous membranes and their suitability for air and water filtrations. Membranes.

[17] Bai Y., Han C.B., He C., Gu G.Q., Nie J.H., Shao J.J., Xiao T.X., Deng C.R., Wang Z.L. (2018). Washable multilayer triboelectric air filter for efficient particulate matter pm2.5removal. Adv. Funct. Mater..

[18] Arifeen W.U., Kim M., Ting D., Kurniawan R., Choi J., Yoo K., Ko T.J. (2020). Hybrid thermal resistant electrospun polymer membrane as the separator of lithium ion batteries. Mater. Chem. Phys..

[19] Liang W., Xu Y., Li X., Wang X.-X., Zhang H.D., Yu M., Ramakrishna S., Long Y.Z. (2019). Transparent polyurethane nanofiber air filter for high-efficiency pm2.5 capture. Nanoscale Res. Lett..

[20] Wang B., Wang Q., Wang Y., Di J., Miao S., Yu J. (2019). Flexible multifunctional porous nanofibrous membranes for high-efficiency air filtration. ACS Appl. Mater. Interfaces.

[21] Liu G., Nie J., Han C., Jiang T., Yang Z., Pang Y., Xu L., Guo T., Bu T., Zhang C. (2018). Self-powered electrostatic adsorption face mask based on a triboelectric nanogenerator. ACS Appl. Mater. Interfaces.

[22] Liao Y., Tian Y., Wang R., Tian M., Huang J.J. (2020). Fabrication of bead-on-string polyacrylonitrile nanofibrous air filters with superior filtration efficiency and ultralow pressure drop. Sep. Purif. Technol..

[23] Das D., Waychal A. (2016). On the triboelectrically charged nonwoven electrets for air filtration. J. Electrostat.

[24] Liu J., Zhang H., Gong H., Zhang X., Wang Y., Jin X. (2019). Polyethylene/polypropylene bicomponent spunbond air filtration materials containing magnesium stearate for efficient fine particle capture. ACS Appl. Mater. Interfaces.

[25] Li X., Wang N., Fan G., Yu J., Gao J., Sun G., Ding B. (2015). Electreted polyetherimide-silica fibrous membranes for enhanced filtration of fine particles. J. Colloid Interface Sci..

[26] Wang S., Zhao X., Yin X., Yu J., Ding B. (2016). Electret polyvinylidene fluoride nanofibers hybridized by polytetrafluoroethylene nanoparticles for high-efficiency air filtration. ACS Appl. Mater. Interfaces.

[27] Yu J., Hou X., He J., Cui M., Wang C., Geng W., Mu J., Han B., Chou X. (2020). Ultra-flexible and high-sensitive triboelectric nanogenerator as electronic skin for self-powered human physiological signal monitoring. Nano Energy.

[28] Khalid S., Raouf I., Khan A., Kim N., Kim H.S. (2019). A review of human-powered energy harvesting for smart electronics: Recent progress and challenges. Int. J. Precis. Eng. Manuf.-Green Tech..

[29] Wu C.-M., Chou M.-H., Zeng W.-Y. (2018). Piezoelectric response of aligned electrospun polyvinylidene fluoride/carbon nanotube nanofibrous membranes. Nanomaterials.

[30] Zhang T., Zhao D., Wang L., Meng R., Zhao H., Zhou P., Xia L., Zhong B., Wang H., Wen G. (2020). A facile precursor pyrolysis route to bio-carbon/ferrite porous architecture with enhanced electromagnetic wave absorption in s-band. J. Alloys Compd..

[31] Wang J., Xia K., Liu J., Li T., Zhao X., Shu B., Li H., Guo J., Yu M., Tang W. (2020). Self-powered silicon pin photoelectric detection system based on triboelectric nanogenerator. Nano Energy.

[32] Gu G.Q., Han C.B., Tian J.J., Jiang T., He C., Lu C.X., Bai Y., Nie J.H., Li Z., Wang Z.L. (2018). Triboelectric nanogenerator enhanced multilayered antibacterial nanofiber air filters for efficient removal of ultrafine particulate matter. Nano Res..

[33] Davies D. (1969). Charge generation on dielectric surfaces. J. Phys. D.

[34] Feng Y., Ling L., Nie J., Han K., Chen X., Bian Z., Li H., Wang Z.L. (2017). Self-powered electrostatic filter with enhanced photocatalytic degradation of formaldehyde based on built-in triboelectric nanogenerators. ACS Nano.

